# Changes in N-glycans of IgG4 and its relationship with the existence of hypocomplementemia and individual organ involvement in patients with IgG4-related disease

**DOI:** 10.1371/journal.pone.0196163

**Published:** 2018-04-19

**Authors:** Naoki Konno, Mitsuru Sugimoto, Tadayuki Takagi, Makiko Furuya, Tomoyuki Asano, Shuzo Sato, Hiroko Kobayashi, Kiyoshi Migita, Yoshiaki Miura, Taichi Aihara, Atsushi Komatsuda, Hiromasa Ohira, Hiroshi Watanabe

**Affiliations:** 1 Department of Gastroenterology, School of Medicine, Fukushima Medical University, Fukushima, Japan; 2 Department of Rheumatology, School of Medicine, Fukushima Medical University, Fukushima, Japan; 3 S-Bio, Sumitomo Bakelite Co., Ltd., Hudson, New Hampshire, United States of America; 4 Department of Hematology, Nephrology, and Rheumatology, School of Medicine, Akita University, Akita, Japan; Emory University School of Medicine, UNITED STATES

## Abstract

**Background:**

Although increased serum IgG4 level and tissue infiltration of IgG4-positive cells are key events in IgG4-related disease (IgG4RD), and nearly half of IgG4RD patients show hypocomplementemia, the role of IgG4 in the pathogenesis of IgG4RD remains unclear. Many reports show that altered IgG glycosylation, especially IgG with agalactosylated N-linked glycan (G0 N-glycan), have proinflammatory roles including complement activation, implicated in the pathogenesis of various inflammatory diseases. This study determined the concentration of N-linked glycans (N-glycan) released from serum IgG4 in IgG4RD patients and compared the difference of glycosylation changes to those in healthy controls. We also compared the concentration of each IgG4 glycoform between patients with and without hypocomplementemia and individual organ involvement (kidney, pancreas, lymph node) in IgG4RD.

**Methods:**

We collected sera from 12 IgG4RD patients and 8 healthy controls. IgG4 was isolated from sera via Melon™ Gel IgG Spin Purification Kit followed by Capture Select IgG4 (Hu) Affinity Matrix. IgG4 N-glycans were analyzed by S-BIO Glycan*Map*^®^ Xpress methodology.

**Results:**

Significant increases of IgG4 G0 N-glycan and IgG4 fucosylated N-glycan (F1 N-glycan) concentrations were observed in IgG4RD compared with healthy controls. Although we observed decreased levels of IgG4 F0 glycan in IgG4RD with hypocomplementemia, there were no significant differences in the galactosylation and sialyation of IgG4 N-glycans. Furthermore, there were no significant differences in the glycosylation of IgG4 N-glycans between patients with and without individual organ involvement of IgG4RD.

**Conclusions:**

Although IgG4 has anti-inflammatory properties, IgG4 G0 and F1 glycans were increased in patients with IgG4RD. Our results suggest that decreased galactosylation of IgG4 is not related to complement activation and the differences of individual organ involvement in IgG4RD. IgG4 fucosylation change may be related to complement activation in IgG4RD. Further investigation is needed to clarify the role of IgG4 in IgG4RD.

## Introduction

IgG4-related disease (IgG4RD) is an emerging clinical disorder characterized by increased serum IgG4 concentrations, dense lymphoplasmacytic infiltrate, IgG4-positive plasma cells, and storiform fibrosis [1, 2]. IgG4RD includes various diseases formerly diagnosed as Mikulicz's Disease [3, 4], autoimmune pancreatitis [5, 6], retroperitoneal fibrosis [7, 8], tubulointerstitial nephritis [9–11], Riedel’s thyroiditis [12], and inflammatory pseudotumor [13]. Although elevated serum IgG4 levels and infiltration of IgG4-plasma cells in tissues are observed, much remains unknown about the role of IgG4 in the pathogenesis of IgG4RD.

Among the subclasses of IgG, IgG4 is the least abundant subclass in human serum. IgG4 is distinguished from other IgG subclasses by its unique functional and structural properties. IgG4 lacks the ability to activate the classical complement pathway because of reduced binding capacity to complement component 1q (C1q)[14–17]. IgG4 also has a low binding capacity for Fcγ receptors (FcγRs)[18] and can exchange half its molecules *in vivo* to form antibodies with two different binding specificities that are monovalent [19].

Although IgG4 itself does not activate the classical complement pathway because of a low C1q binding capacity, nearly half of IgG4RD patients show hypocomplementemia[11, 20]. Several reports showed the association of immune complexes (ICs) containing IgG1 with hypocomplementemia, and suggested that complement activation in IgG4RD is caused by ICs containing IgG1, 2, 3, and IgM, but not IgG4, via the classical pathway[20–22]. However, our previous report showed IC containing IgG4 and IgM, isolated from IgG4RD patients with hypocomplementemia, activated the complement system via the classical pathway as well as the lectin pathway[23]. Thus, the mechanism and the role of IgG4 in complement activation in IgG4RD remains unclear.

Protein glycosylation is a post-translational modification affecting the structures and functions of many proteins required for normal immune function. Over the past decades, many studies have investigated the structural and biological roles of immunoglobulin G (IgG) glycosylation. In IgG molecules, two N-glycans are linked to heavy chains at asparagine (Asn) residues (CH2–84.4) in the CH2 domain of the Fc portion[24, 25]. Fc-linked glycans exhibit complex-type bi-antennary N-glycans with a high content of core fucose and a variable number of galactose residues leading to the major glycoforms G0 (agalactosylated), G1 (monogalactosylated), and G2 (digalactosylated). Some populations of Fc-linked glycans contain bisection GlcNAc and/or terminal sialic acids.

Recent studies revealed that IgG glycosylation, especially the degree of galactosylation, is associated with various physiological and pathological conditions[26]. Clinically, increased levels of serum IgG with G0 glycan were observed in patients with rheumatoid arthritis (RA), Crohn’s disease, systemic lupus erythematosus, or granulomatosis with polyangiitis (GPA, formerly named Wegener’s granulomatosis) [27–29], and elevated levels of IgG with G0 glycans were also correlated with RA disease activity [30]. Furthermore, the lack of core-fucose or the presence of bisecting GlcNAc improved the affinity of Fc binding to FcγR IIIa, resulting in increased antibody-dependent cellular cytotoxicity[31–33]. It was also reported that a lack of sialic acid and low levels of galactosylation promoted proinflammatory functions[34–36]. Interestingly, agalactosylated IgG binds to mannose-binding protein (MBL) in serum and activates the lectin complement pathway, which participates in the pathogenesis of RA[37, 38].

Although recent studies revealed the importance of altered IgG glycosylation in the pathogenesis of various autoimmune and inflammatory diseases, little is known about the glycosylation of IgG4 in IgG4RD. In this study, we determined the occurrence (%) and absolute concentration of each glycoform released from IgG4 isolated from the sera of IgG4RD patients and healthy controls. We also compared the differences of glycosylation changes between patients with IgG4RD and healthy controls. The occurrence (%) and absolute concentration of each glycoform released from IgG4 isolated from the sera of IgG4RD patients were compared between patients with and without hypocomplementemia and individual organ involvement (kidney, pancreas, lymph node) for galactosylation, fucosylation, and sialyation in IgG4RD.

## Materials and methods

### Patients and blood samples

Overall, 12 patients with IgG4 related disease (IgG4RD) who fulfilled the comprehensive diagnostic criteria for IgG4RD[1] and 8 healthy controls were included in this study. Patient demographics are summarized in Table 1. Sera were separated from the blood samples and stored at −80°C until further analysis. Written informed consent was obtained from the patients, next of kin or legally authorized guardians. This study, including the process of securing informed consent, was approved by the ethics Committee of Fukushima Medical University (approval number 1722), which is guided by local policy, national law, and the World Medical Association Declaration of Helsinki.

**Table 1:** Clinical characteristics of patients.

Patient	Age/Gender	IgG4 (mg/dl)	C3/C4 (mg/dl)	CH50	Involvement of organs
1	69/M	768	49/2	<5	Kidney, Pancreas, LN
2	77/F	1160	20/1	<5	Submandibular gland, LN, Lung
3	62/M	1160	59/3	<12	LN, Retroperitoneal fibrosis
4	50/M	2630	37/1	N.D.	LN
5	65/M	297	35/1	<12	Kidney, Pancreas, Sclerosing cholangitis
6	60/M	670	21/2	<12	Kidney, Submandibular gland, LN
7	64/F	1020	22/0.7	7	Kidney
8	60/M	480	120/38	64.8	Pancreas
9	48/F	108	105/34	40.5	Pancreas
10	64/M	945	107/18	41	Submandibular gland, Retroperitoneal fibrosis, Kidney
11	72/M	289	82/18	35	Lung
12	53/M	1040	131/42	54	Submandibular gland, LN, Lung, Kidney

M, male; F, female; N.D., not done; LN, lymph node

### Isolation of IgG from patient sera

IgG was isolated using the Melon™ Gel IgG Spin Purification Kit (Thermo Fisher Scientific, Rockford, IL, USA) according to the manufacturer’s instructions. Briefly, serum was dialyzed with Melon™ Gel purification buffer, and mixed with 20% slurry gel in a spin column. After end-over-end mixing for 5 min at room temperature (RT), the spin column was centrifuged at 5,000 ×*g* for 1 min to collect purified IgG. The concentration of purified IgG was measured by absorbance at 280 nm using an extinction coefficient (*E*_280_^0.1%^) of 1.4.

### Isolation of IgG4

IgG4 was isolated via Capture Select IgG4 (Hu) Affinity Matrix (Life Technologies, Paisley, UK) according to the manufacturer’s instructions. Briefly, purified IgG was loaded onto a 2 ml Capture Select IgG4 column in fully preserved PBS. Then, the column was eluted with 0.1 M glycine-HCl buffer (pH 2.8). The eluted IgG4 fractions were neutralized with 1/10 volume Tris-HCl (pH 9.0). Purified IgG4 was immediately buffer exchanged in ddH_2_O, then concentrated using Amicon ultra-0.5 ml (Ultracel®-3K) (EMD Millipore, Billerica, MA, USA). Protein concentrations were determined using the Bio-Rad Protein Assay (Bio-Rad Laboratories, Inc. Hercules, CA, USA). IgG4 purities were established by SDS-PAGE and western blotting.

### Analyzation of isolated IgG4 purity for IgG subclass composition by western blotting

Isolated IgG4 were analyzed by western blotting assays for IgG subclass composition. Isolated IgG4 were resuspended in non-reduced sample buffer, subjected to 10% SDS-PAGE, and electrophoretically transferred onto a polyvinylidene difluoride (PVDF) membrane. After incubation with blocking buffer, the membrane was incubated separately with biotinylated monoclonal anti-human IgG subclass 1–4 (BD Bioscience, Franklin Lakes, NJ, USA) diluted at 1:1000 in blocking buffer. After incubation at 4°C overnight, the membrane was washed with PBS-T, incubated with Vectastain ABC Reagent (Vector Laboratories, Inc., Burlingame, CA, USA) for 30 min at RT, and subsequently developed with 3,3′-diaminobenzidine tetrahydrochloride. The color of the image was converted from brown to black by computer.

### Glycomic analysis of isolated IgG4

Each sample was analyzed to quantitate N-linked glycans using S-BIO proprietary Glycan*Map®* Xpress methodology based on methods previously reported[39–41]. Several control samples were also analyzed to assess linearity, repeatability, sensitivity, and accuracy. After transferring aliquots of each sample to a 96-well plate and adding internal standards to aid quantitation, samples were denatured, digested with trypsin, and heat-inactivated. N-glycans were then released with PNGase F (New England Biolabs, Ipswich, Massachusetts, USA) and captured chemoselectively on BlotGlyco® beads (Sumitomo Bakelite Co. Ltd., Tokyo, Japan), followed by methyl esterification of the sialic acid residues for stabilization of the mass spectrometric analysis. Finally, glycans were released from the beads, simultaneously labeled, and spotted onto a MALDI target plate. All processes from initial aliquoting to spotting on the MALDI plate were fully automated, using 96-well format robotic technology.

MALDI-TOF mass spectrometry (MS) analysis was performed on an ultraflex III mass spectrometer (Bruker Daltonics, Yokohama, Japan) in the positive-ion, reflectron mode using S-BIO proprietary matrix composition. Each sample was spotted in quadruplicate, and spectra were obtained in an automated manner using the AutoXecute feature in flexControl software (Bruker Daltonics, Yokohama, Japan).

Resulting mass spectra were analyzed using S-BIO proprietary bioinformatic programs, which correct for mass differences induced by the chemical derivatization inherent in the Glycan*Map*® Xpress assay.

Glycan compositions were assigned to each peak based on m/z. The intensities from each of the four quadruplicated spectra were normalized to that of an internal standard with a known concentration and averaged. Data were also corrected for the inherent decrease in peak intensity as m/z increases in mass spectrometric measurements, yielding absolute concentrations that are comparable to those created by HPLC/fluorescent methods. Glycans representing less than 0.5% of the total glycan concentration were not reported.

Glycan compositions were shown as a code of five digit. The code represents the number of hexose (Galactose (Gal), Mannose (Man), or Glucose (Glc)), N-acetylhexosamine (N-acetylglucosamine (GlcNAc) or N-acetylgalactosamine (GalNAc)), deoxyhexose (Fucose (Fuc)), N-acetylneuraminic acid (N-acetylneuraminic acid (Neu5Ac)), and N-glycolylneuraminic acid (N-glycolylneuraminic acid (Neu5Gc)) residues.

### Statistical analysis

Statistical analysis was performed using the Mann-Whitney *U*-test for the comparison of median values. Statistical analyses were performed using SPSS software, version 22 (IBM SPSS, Armonk, NY). *P* values less than 0.05 were considered statistically significant.

## Results

### Purity of IgG4 isolated from sera of IgG4-related disease patients

**Fig 1:**
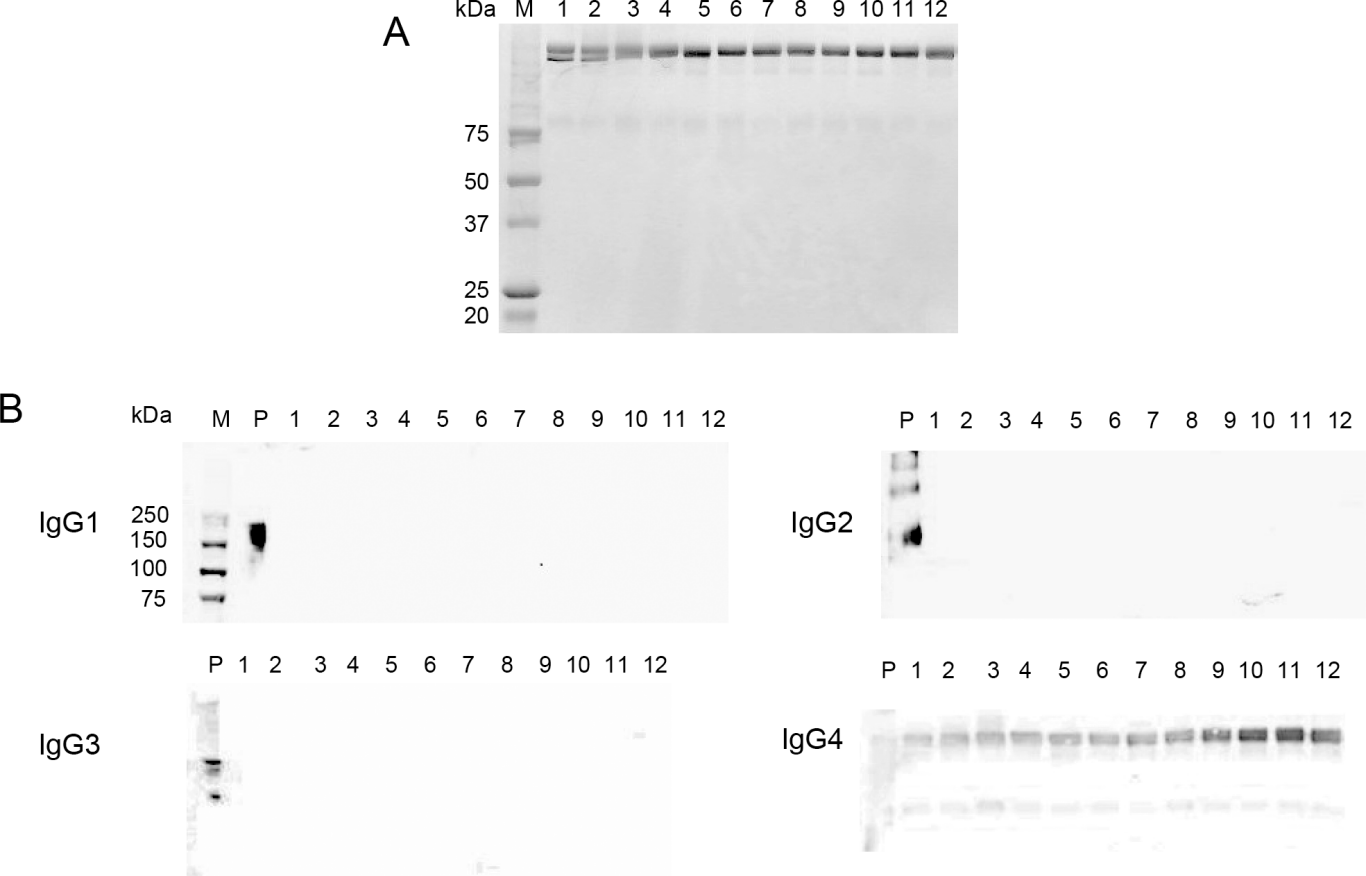
IgG4 isolated from the sera of patients with IgG4-related disease. (A) SDS-PAGE analysis of isolated IgG4. Bands of marker proteins over 75 kDa are blurry and unclear. (B) Western blot analysis of anti-IgG subclasses reacting with isolated IgG4. Blot reactivities for anti IgG4 antibody were observed in all samples.

The purity of IgG4 isolated from the sera of patients with IgG4RD was confirmed by SDS-PAGE and western blot analysis. We did not observe protein contamination by SDS-PAGE (Fig 1A) (S1 Fig). Blot reactivity was only observed for anti-IgG4 antibody to isolated IgG4 from the sera of IgG4RD patients (Fig 1B) (S2–S5 Figs).

### Characterization of glycans of IgG4

**Fig 2:**
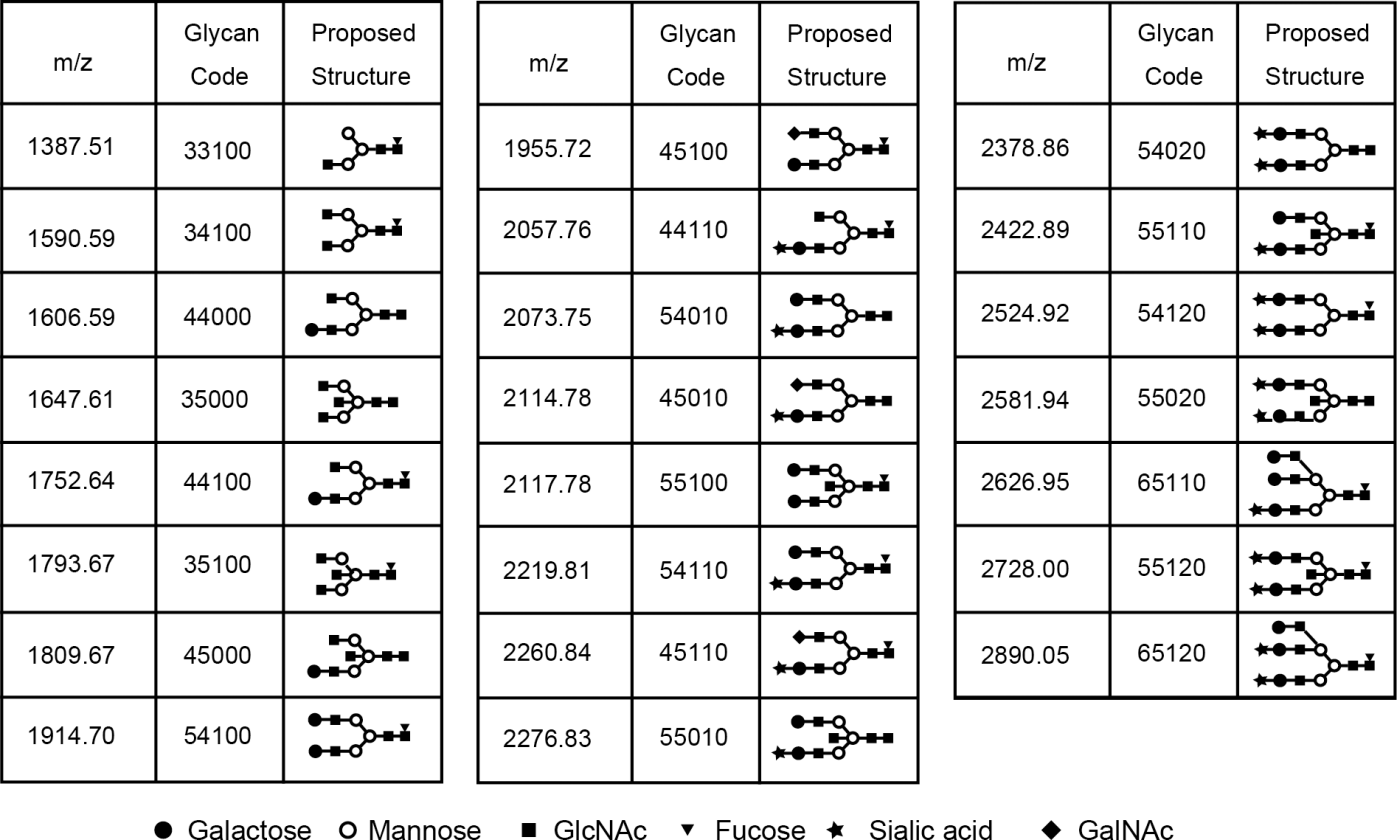
Glycan codes and proposed structures for 23 glycans detected in this study.

We detected 23 glycans released from isolated IgG4 (S1 Data). Proposed structures for all 23 glycans and the code used are shown in Fig 2. Four glycans were agalactosylated (G0), 7 glycans were monogalactosylated (G1), 10 glycans were digalactosylated (G2), and 2 glycans were trigalactosylated (G3). Fourteen glycans were fucosylated (F1), and 9 glycans were afucosylated (F0). Eleven glycans were asialylated (S0), 8 glycans were monosialylated (S1), and 4 glycans were disialylated (S2). Seven glycans had a bisecting arm (B1), and 17 glycans did not (B0).

### Occurrence of each glycan group (G0, G1, G2, G3, F0, F1, S0, S1, S2, B0, B1) released from IgG4 isolated from the sera of patients with IgG4-related disease and healthy controls

Results are expressed as the percentage of each glycan group released from IgG4 isolated from the sera of IgG4RD patients and healthy controls. As shown in Table 2, no significant differences in the percentage of each glycan group (G0, G1, G2, G3, F0, F1, S0, S1, S2, B0, B1) were observed when IgG4RD and healthy controls were compared.

**Table 2:** Occurrence of each glycan group released from IgG4.

	IgG4RD (n = 12) Median (range)	Healthy control (n = 8) Median (range)	p value
G0 (%)	36.3 (30.7–48.2)	35.3 (19.9–48.7)	NS
G1 (%)	23.8 (21.6–27.2)	27.2 (23.3–30.3)	NS
G2 (%)	38.2 (27.7–45.5)	37.3 (28.0–51.1)	NS
G3 (%)	0.5 (0.0–1.1)	0.0 (0.0)	NS
F0 (%)	3.7 (2.0–9.6))	6.5 (2.9–8.1)	NS
F1 (%)	96.3 (90.4–98.0)	93.6 (91.9–97.1)	NS
S0 (%)	61.8 (52.8–69.7)	67.6 (59.5–72.2)	NS
S1 (%)	23.3 (19.5–27.5)	20.7 (17.3–23.3)	NS
S2 (%)	14.6 (10.7–21.2)	11.7 (9.5–17.2)	NS
B0 (%)	75.6 (68.3–85.6)	80.0 (76.3–84.3)	NS
B1 (%)	24.4 (14.4–31.7)	20.0 (15.7–23.7)	NS

IgG4RD, IgG4-related disease; NS, not significant.

### Absolute concentration of each glycome released from IgG4 isolated from sera of patients with IgG4-related disease and healthy controls

#### Galactosylation of IgG4 glycome

**Fig 3:**
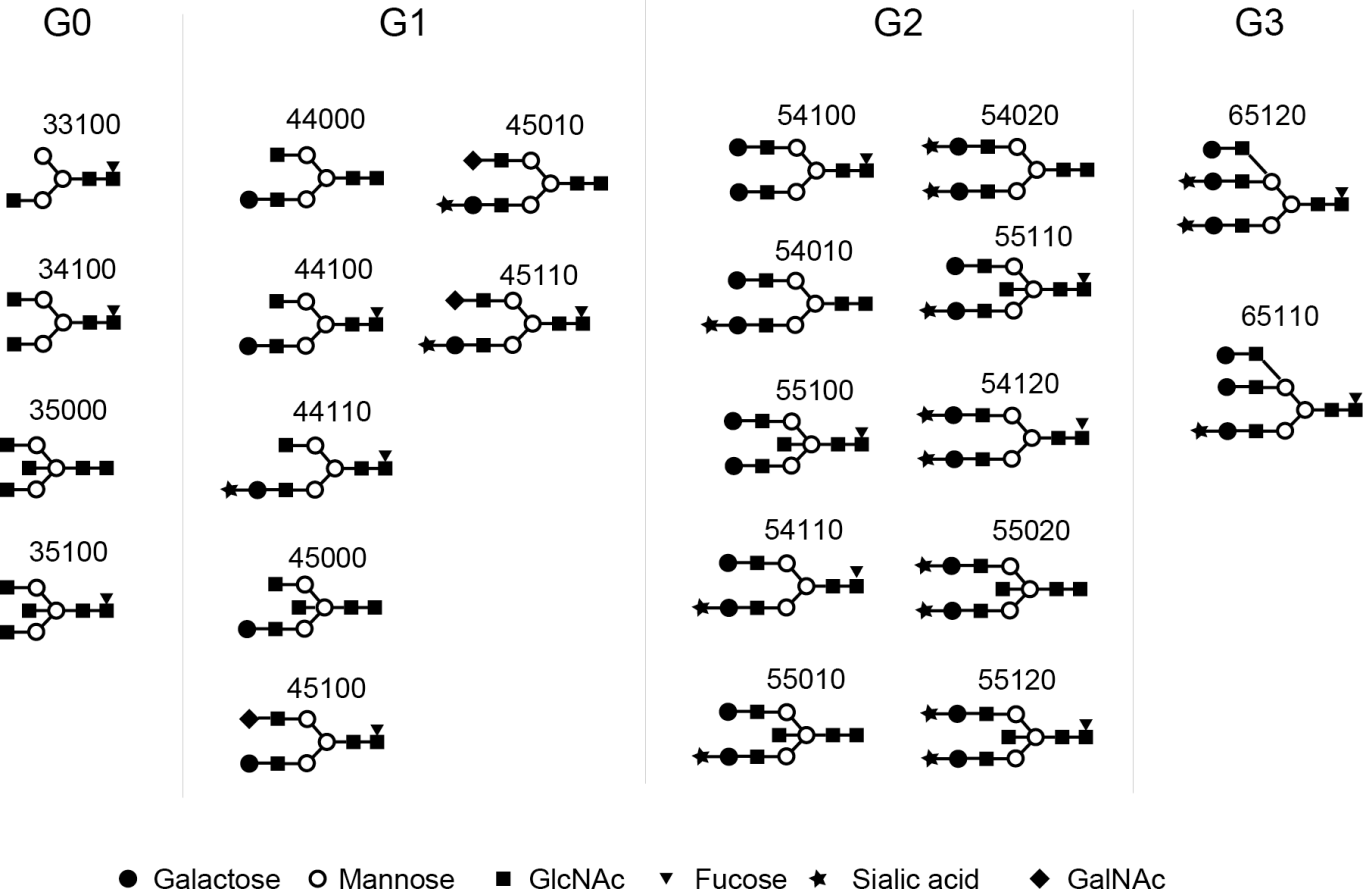
**Structures of agalactosylated (G0), monogalactosylated (G1), digalactosylated (G2), and trigalactosylated (G3) glycans released from IgG4 isolated from the sera of patients with IgG4RD and healthy controls.** GlcNAc: N-Acetylglucosamine, GalNAc: N-Acetylgalactosamine.

**Fig 4:**
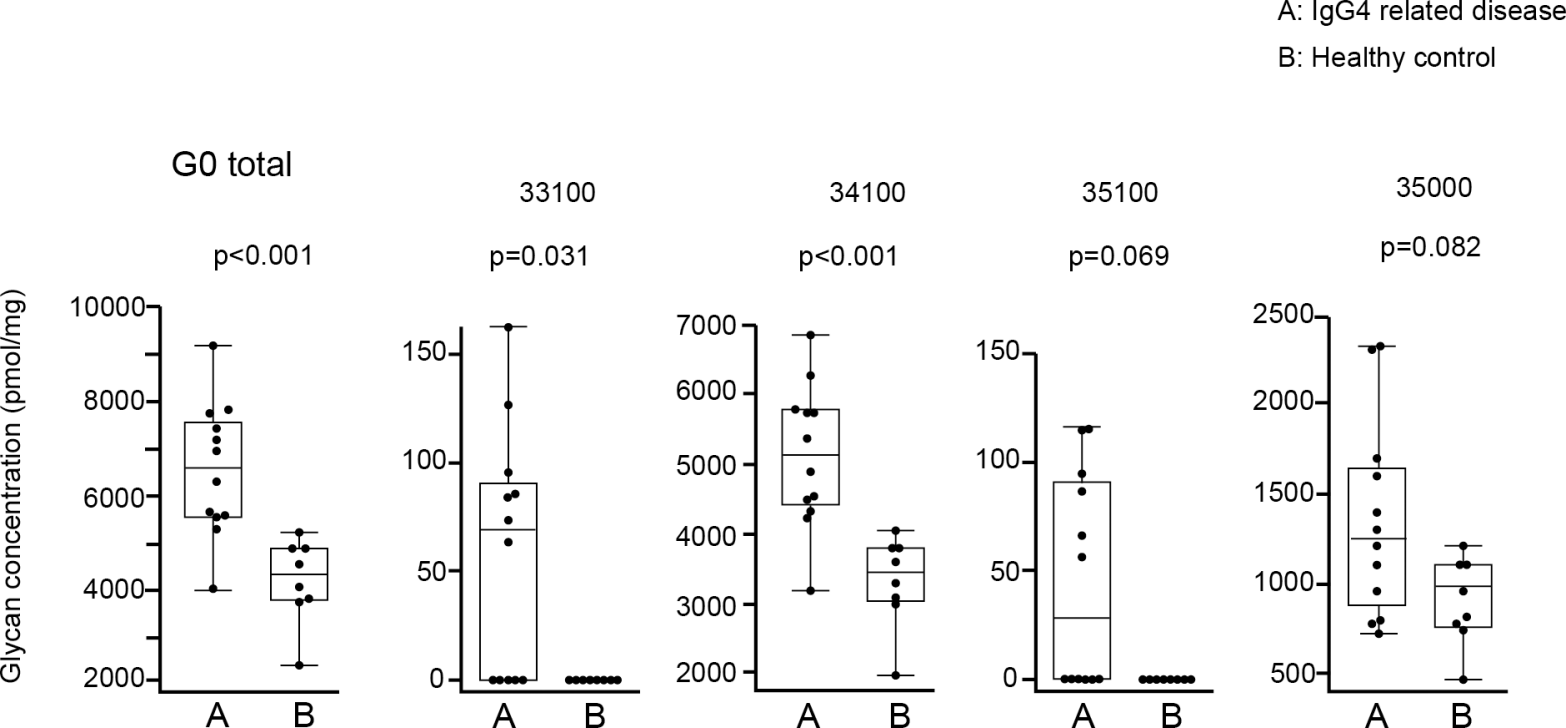
Comparison of absolute concentrations of IgG4 G0 glycans between IgG4RD and healthy controls. Data are shown as box plots. The boxes indicate the upper and lower interquartile range (IQR), the lines within the boxes indicate the median, and the whiskers indicate the minimum and maximum IQR. Each dot represents an individual sample. Statistical analysis was performed using the Mann-Whitney *U*-test.

Based on the number of galactoses, N-glycans released from IgG4 (IgG4 glycan) were divided into 4 groups: glycans without galactose (G0 IgG4 glycan), with monogalactose (G1 IgG4 glycan), with digalactose (G2 IgG4 glycan), and with trigalactose (G3 IgG4 glycan) (Fig 3). Absolute concentrations of IgG4 glycan in each group were compared between IgG4RD and healthy controls. As shown in Fig 4, 2 of 4 G0 IgG4 glycans (33100, 35100) were unique in the IgG4 of IgG4RD. Significant elevations of G0 IgG4 glycan levels were observed in 2 of 4 G0 IgG4 glycans (33100, 34100). Total G0 IgG4 glycan level was also significantly increased in IgG4RD compared with healthy controls.

**Fig 5:**
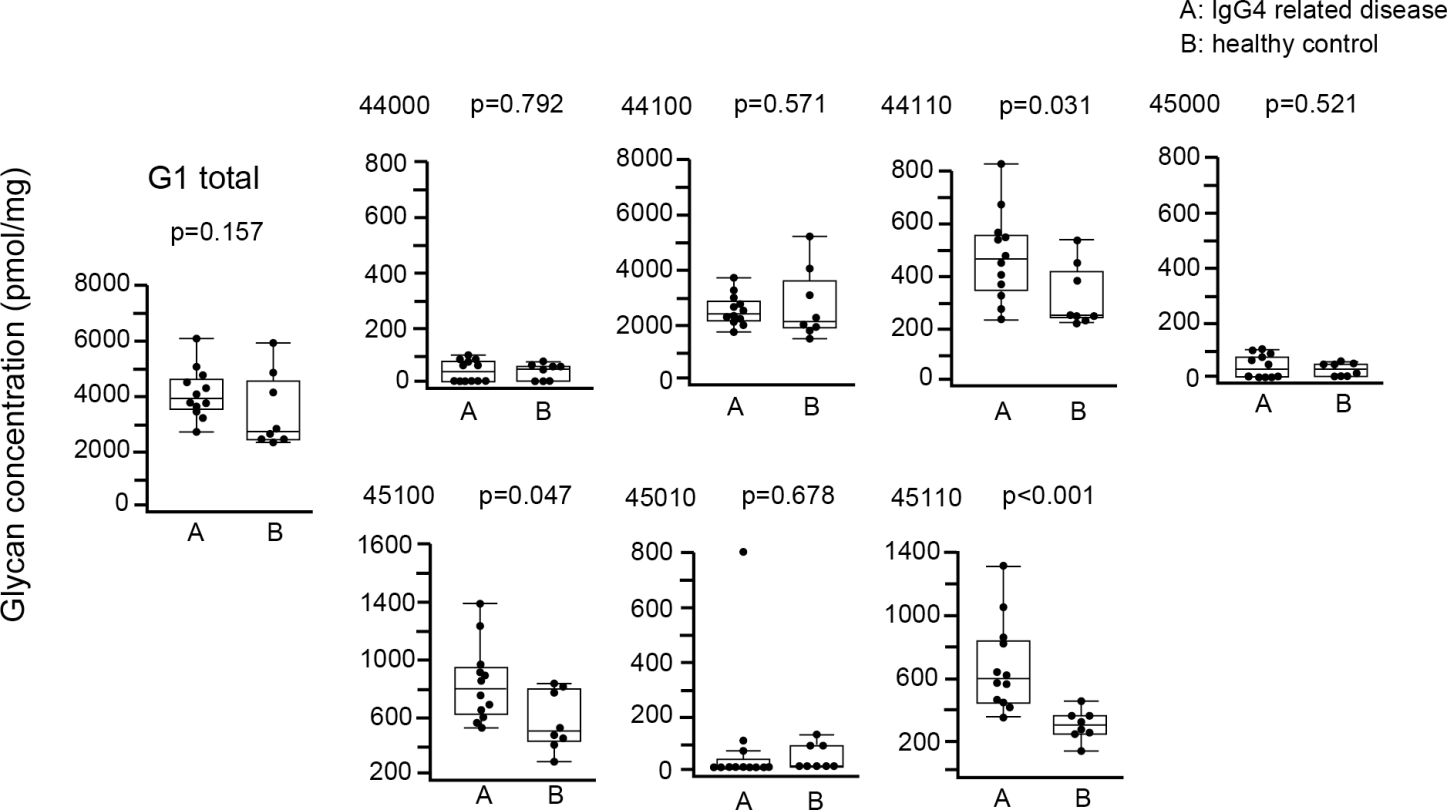
Comparison of absolute concentration of IgG4 G1 glycans between IgG4RD and healthy controls. Data are shown as box plots. The boxes indicate the upper and lower interquartile range (IQR), the lines within the boxes indicate the median, and the whiskers indicate the minimum and maximum IQR. Each dot represents an individual sample. Statistical analysis was performed using the Mann-Whitney *U*-test.

Regarding G1 IgG4 glycans, there was no significant difference in total G1 IgG4 glycan between IgG4RD and healthy controls, although 3 of 7 G1 IgG4 glycans (44110, 45100, 45110) were significantly increased in IgG4RD compared with healthy controls (Fig 5).

**Fig 6:**
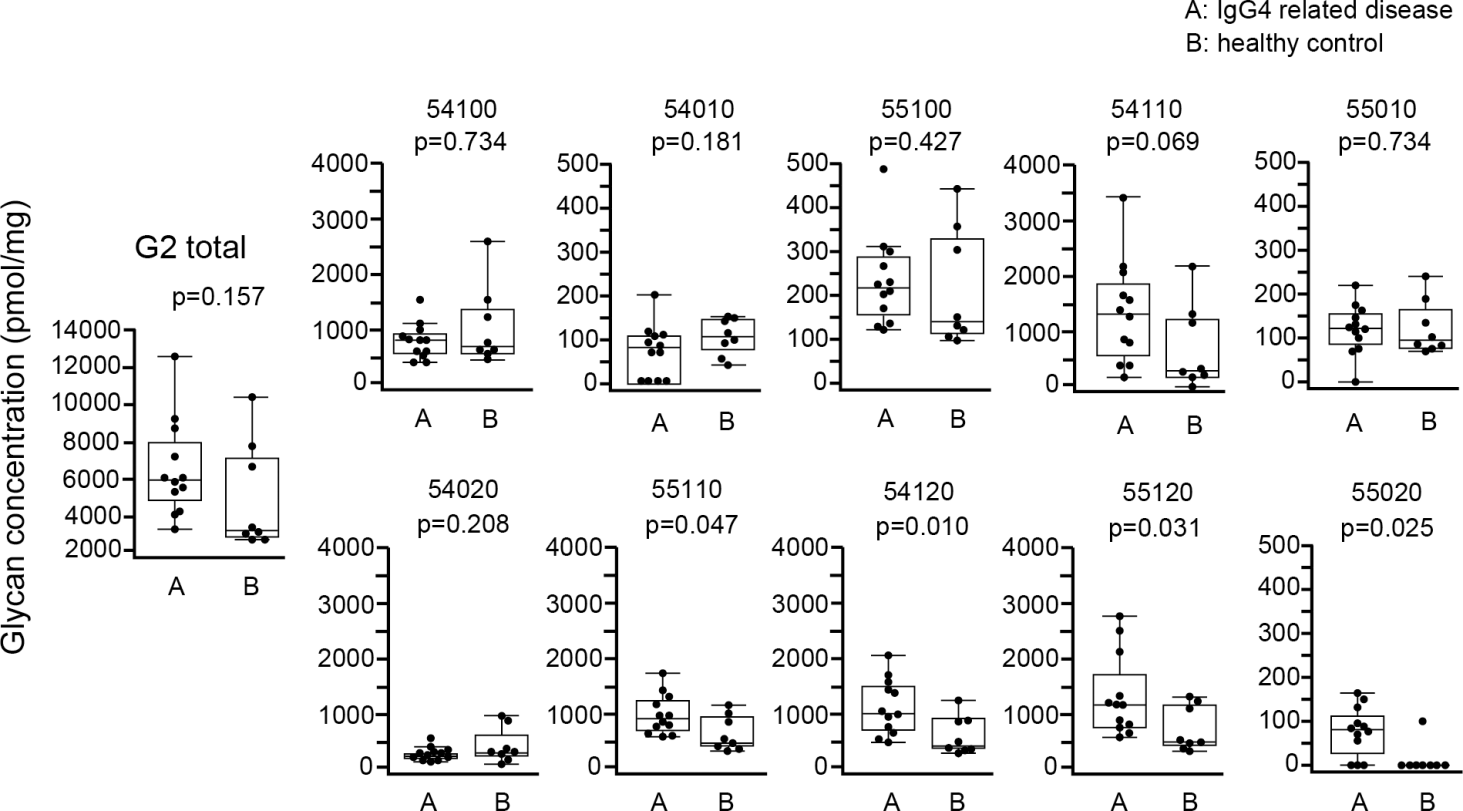
Comparison of absolute concentration of IgG4 G2 glycans between IgG4RD and healthy controls. Data are shown as box plots. The boxes indicate the upper and lower interquartile range (IQR), the lines within the boxes indicate the median, and the whiskers indicate the minimum and maximum IQR. Each dot represents an individual sample. Statistical analysis was performed using the Mann-Whitney *U*-test.

Regarding G2 IgG4 glycans, there was no significant difference in total G2 IgG4 glycan between IgG4RD and healthy controls, although 4 of 10 G2 IgG4 glycans (55110, 54120, 55120, 55020) were significantly increased in IgG4RD compared with healthy controls (Fig 6).

**Fig 7:**
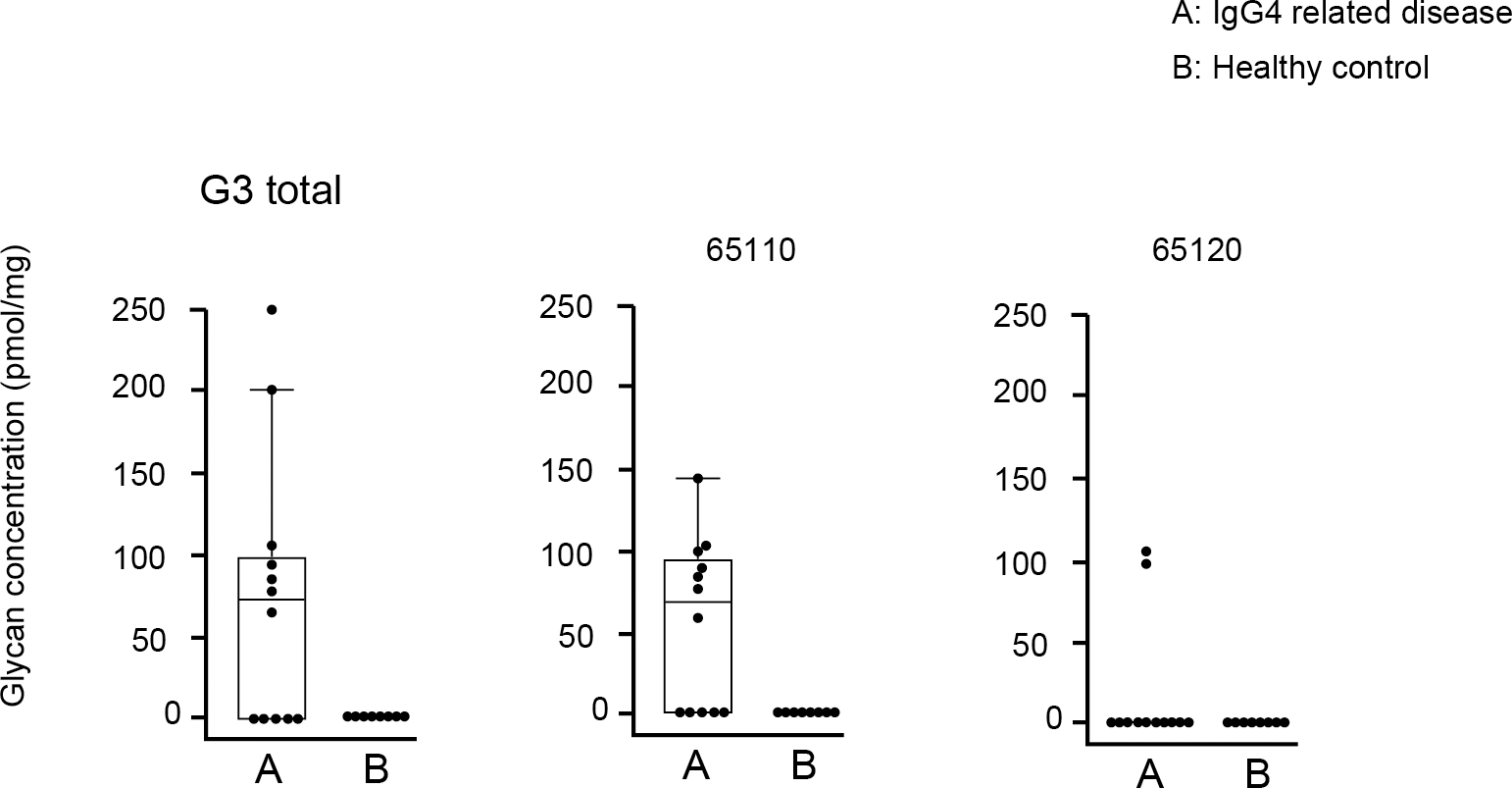
Comparison of absolute concentration of IgG4 G0 glycans between IgG4RD and healthy controls. Data are shown as box plots. The boxes indicate the upper and lower interquartile range (IQR), the lines within the boxes indicate the median, and the whiskers indicate the minimum and maximum IQR. Each dot represents an individual sample. Statistical analysis was not performed.

As shown in Fig 7, although present at low concentrations, G3 IgG4 glycans (65110, 65120) were unique in IgG4RD. We did not perform statistical analysis for G3 IgG4 glycans because of the small number of samples.

#### Fucosylation of IgG4 glycan

**Fig 8:**
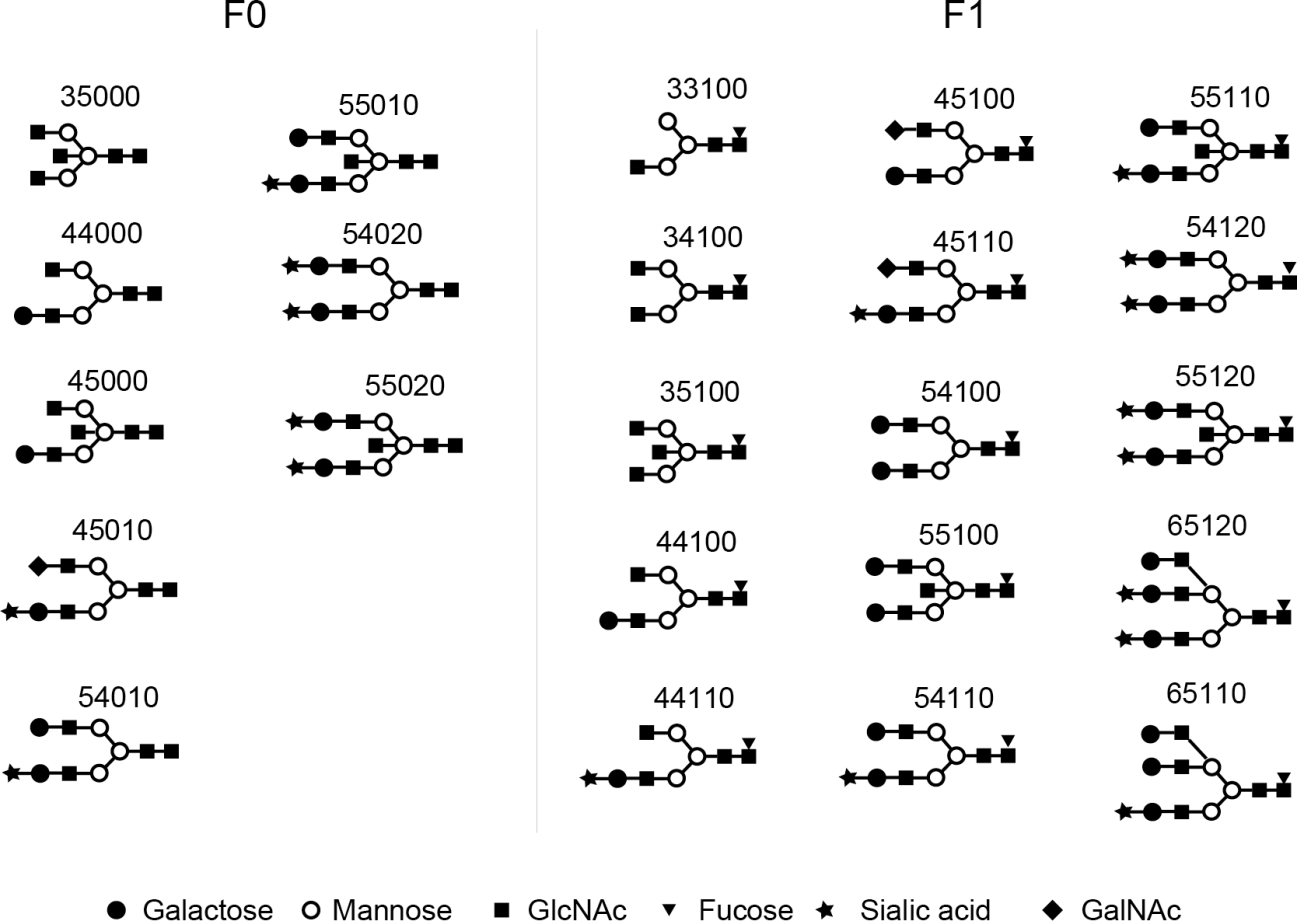
**Structures of afucosylated (F0) and fucosylated (F1) glycans released from IgG4 isolated from the sera of patients with IgG4RD and healthy controls.** GlcNAc: N-Acetylglucosamine, GalNAc: N-Acetylgalactosamine.

**Fig 9:**
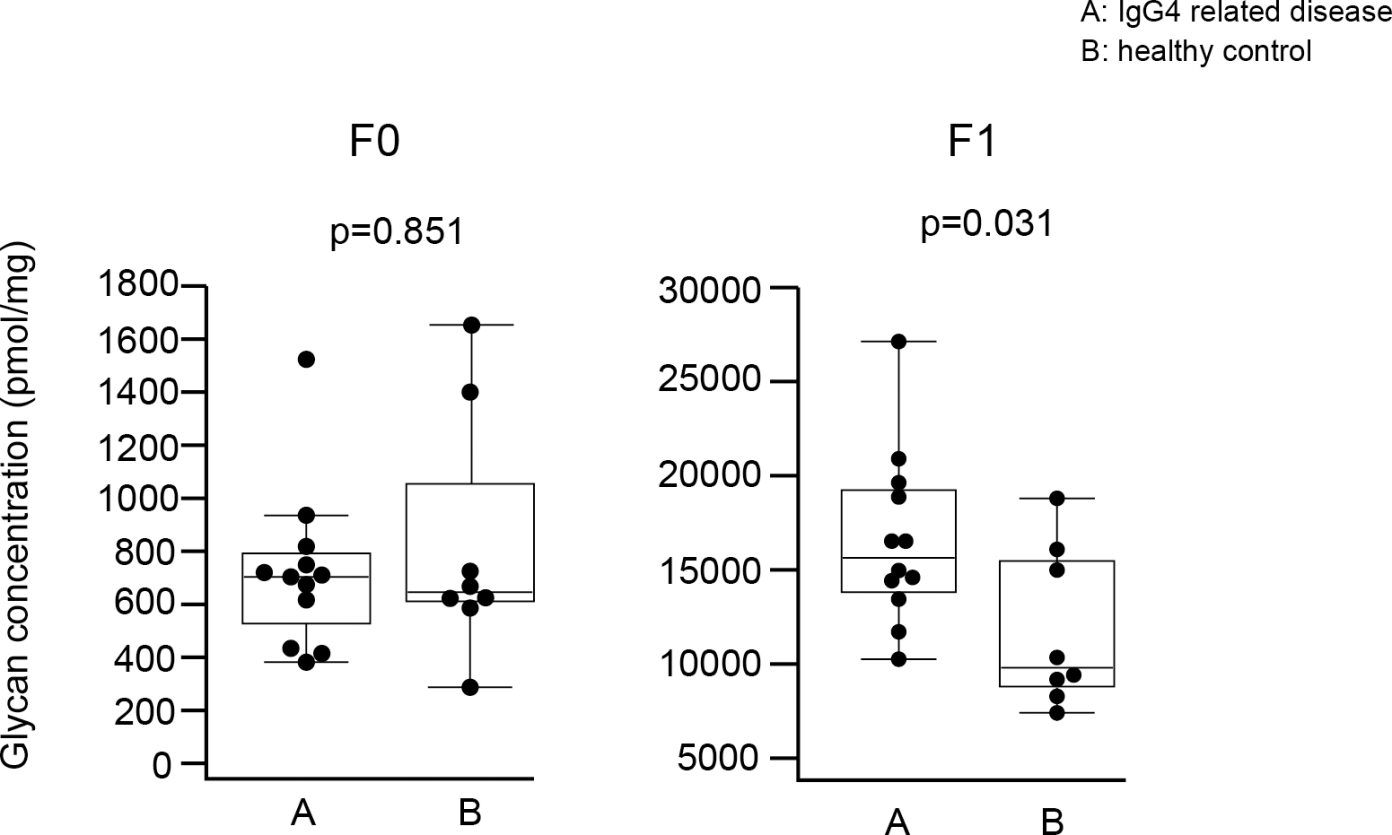
Comparison of absolute concentration of IgG4 F0 and F1 glycans between IgG4RD and healthy controls. Data are shown as box plots. The boxes indicate the upper and lower interquartile range (IQR), the lines within the boxes indicate the median, and the whiskers indicate the minimum and maximum IQR. Each dot represents an individual sample. Statistical analysis was performed using the Mann-Whitney *U*-test.

Based on the number of fucoses, IgG4 glycans were divided into 2 groups: glycans without fucose (F0 IgG4 glycan) and with fucose (F1 IgG4 glycan) (Fig 8). As shown in Fig 9, F1 IgG4 glycan levels were increased in IgG4RD compared with healthy controls. However, there was no significant difference in the concentration of F0 glycans between IgG4RD and healthy controls.

#### Sialylation of IgG4 glycan

**Fig 10:**
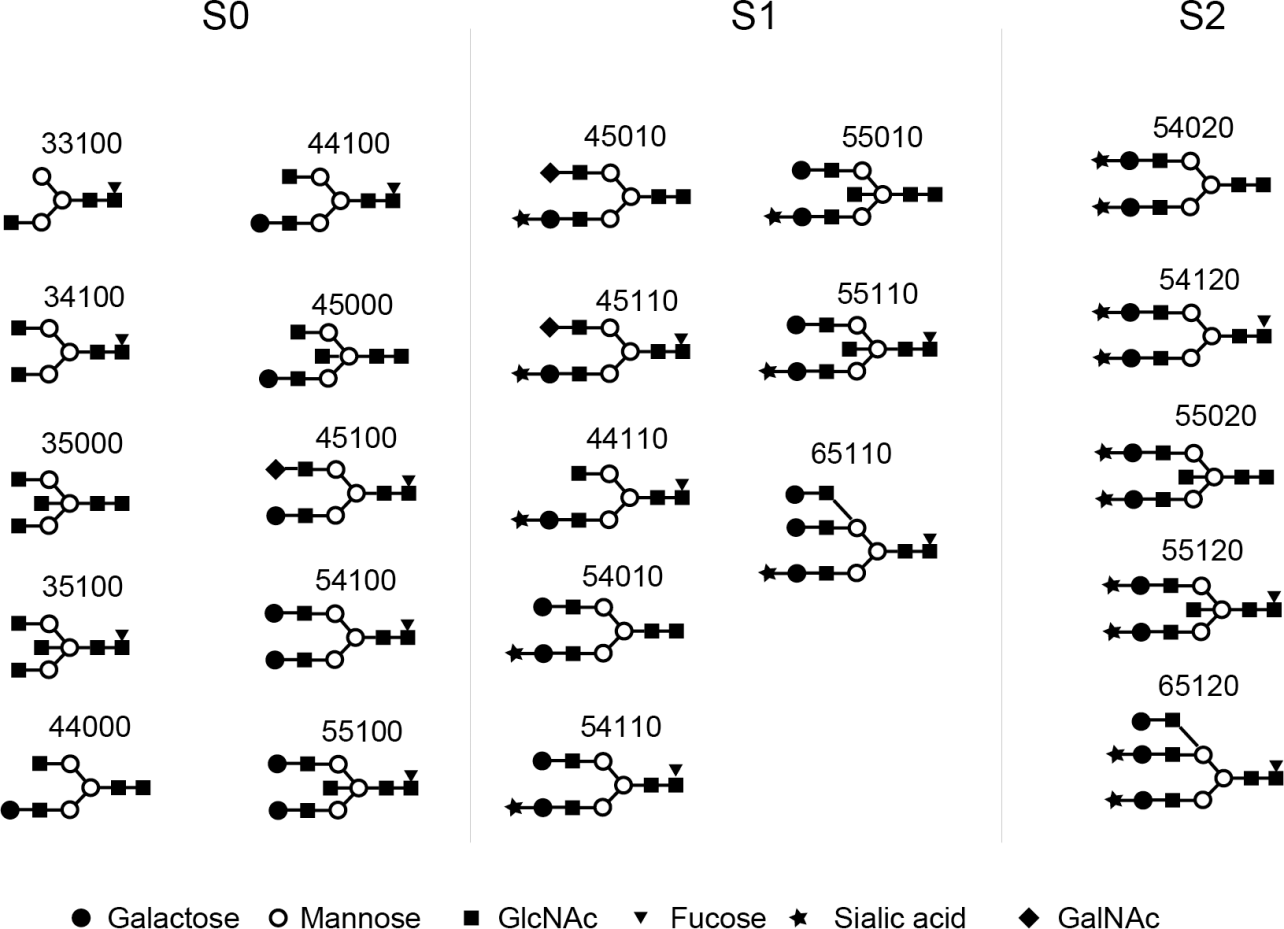
**Structures of asialylated (S0), monosialylated (S1), and disialylated (S2) glycans released from IgG4 isolated from the sera of patients with IgG4RD and healthy controls.** GlcNAc: N-Acetylglucosamine, GalNAc: N-Acetylgalactosamine.

**Fig 11:**
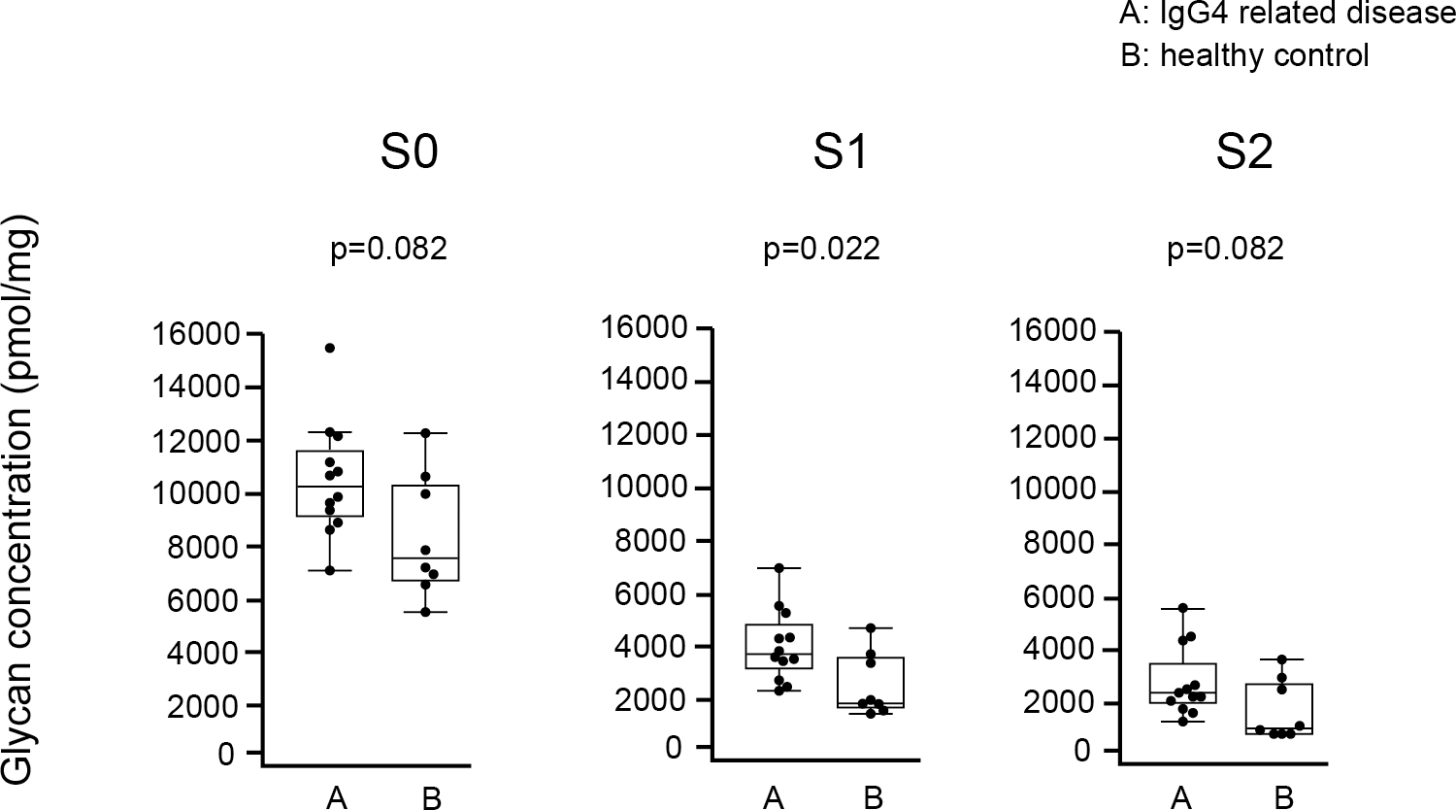
**Comparison of absolute concentration of IgG4 S0, S1, and S2 glycans between IgG4RD and healthy controls.** Data are shown as box plots. The boxes indicate the upper and lower interquartile range (IQR), the lines within the boxes indicate the median, and the whiskers indicate the minimum and maximum IQR. Each dot represents an individual sample. Statistical analysis was performed using the Mann-Whitney *U*-test.

Based on the number of sialic acids, glycans detected in IgG4 were divided into 3 groups: glycans without sialic acid (S0 IgG4 glycan), with 1 sialic acid (S1 IgG4 glycan), and with 2 sialic acids (S2 IgG4 glycan) (Fig 10). As shown in Fig 11, concentrations of S1 glycan were significantly elevated in IgG4RD compared with healthy controls. There were no significant differences in the concentrations of S0 and S2 IgG4 glycans between IgG4RD and healthy controls.

### The relationship between manifestations of IgG4RD and IgG4 glycan composition

IgG4 glycan level was compared between patients with and without hypocomplementemia and for individual organ involvement (kidney, pancreas, lymph node) for galactosylation, fucosylation, and sialyation in IgG4RD. As shown in Table 3, there was a significant difference in F0 IgG4 glycans in IgG4RD with hypocomplementemia compared with those without hypocomplementemia. However, no significant differences in the galactosylation, fucosylation, and sialyation of IgG4 glycans were observed when IgG4RD with and without other individual organ involvement (kidney, pancreas, lymph node) were compared (Tables 4, 5 and 6).

**Table 3:** Comparison of the percentage and concentration of each IgG4 N-glycan between patients with IgG4-related disease with or without hypocomplementemia.

	Hypocomplementemia (+) Patients 1–7 (n = 7) Median (range)	Hypocomplementemia (−) Patients 8–12 (n = 5) Median (range)	p value
G0 (%)	36.1 (30.7–44.7)	36.6 (32.8–48.2)	0.876
G0 (pmol/mg)	7204.8 (5322.8–7817.3)	5641.9 (4039.6–9210.5)	0.432
G1 (%)	23.8 (22.5–24.3)	24.7 (21.6–27.2)	0.530
G1 (pmol/mg)	4048.8 (3694.5–5036.2)	3434.9 (2724.5–6065.8)	0.343
G2 (%)	39.3 (32.2–45.5)	37.5 (27.7–45.5)	0.343
G2 (pmol/mg)	6201.3 (5542.9–9337.7)	4283.0 (3505.8–12763.2)	0.202
G3 (%)	0.5 (0.0–1.1)	0.5 (0.0–0.7)	0.876
G3 (pmol/mg)	74.7 (0.0–251.2)	64.0 (0.0–102.5)	0.755
F0 (%)	3.5 (2.0–4.4)	6.5 (2.8–9.6)	0.030
F0 (pmol/mg)	626.8 (394.8–4.4)	784.2 (722.6–1537.1)	0.030
F1 (%)	96.5 (95.6–98.0)	93.5 (90.4–97.2)	0.030
F1 (pmol/mg)	16,583.9 (14,760.6–21,205.5)	13,592.5 (10,324.5–27,255.3)	0.106
S0 (%)	61.0 (52.8–64.8)	63.5 (55.7–69.7)	0.343
S0 (pmol/mg)	10,824.6 (8952.8–12,251.5)	9343.9 (7009.4–15,631.6)	0.268
S1 (%)	23.7 (21.1–26.1)	22.0 (19.5–27.5)	0.530
S1 (pmol/mg)	3854.4 (3600.0–5552.0)	2835.0 (2432.1–7002.6)	0.343
S2 (%)	15.4 (13.7–21.2)	14.0 (10.7–19.3)	0.202
S2 (pmol/mg)	2622.0 (2412.5–4374.0)	1930.0 (1358.1–5405.3)	0.106

**Table 4:** Comparison of the percentage and concentration of each IgG4 N-glycan between patients with IgG4-related disease with or without autoimmune pancreatitis.

	Autoimmune pancreatitis (+) n = 4 Median (range)	Autoimmune pancreatitis (−) n = 8 Median (range)	p value
G0 (%)	35.8 (35.09–44.7)	37.3 (30.7–48.2)	NS
G0 (pmol/mg)	5458.4 (4039.6–7698.2)	7079.9 (5641.9–9210.5)	NS
G1 (%)	24.5 (22.5–27.0)	23.3 (21.6–27.2)	NS
G1 (pmol/mg)	3786.5 (2724.5–4318.8)	4328.0 (3240.0–6065.8)	NS
G2 (%)	38.1 (32.2–40.6)	38.5 (27.7–45.5)	NS
G2 (pmol/mg)	5765.3 (4283.5–6157.5)	6750.1 (3505.8–12763.2)	NS
G3 (%)	0.3 (0–0.5)	0.5 (0–1.1)	NS
G3 (pmol/mg)	40.9 (0–91.1)	69.3 (0–251.2)	NS
F0 (%)	5.1 (2.7–9.6)	3.6 (2.0–6.5)	NS
F0 (pmol/mg)	674.7 (414.2–1537.1)	735.0 (394.8–942.5)	NS
F1 (%)	94.9 (90.4–97.3)	96.4 (93.5–98.0)	NS
F1 (pmol/mg)	14602.9 (10324.5–16583.9)	17751.7 (11901.2–27255.3)	NS
S0 (%)	61.2 (58.5–64.8)	61.8 (52.8–69.7)	NS
S0 (pmol/mg)	9148.3 (7009.4–11160.7)	10737.9 (8817.4–15631.6)	NS
S1 (%)	22.9 (21.1–27.5)	23.3 (19.5–26.1)	NS
S1 (pmol/mg)	3618.8 (2432.1–4393.2)	4157.1 (2470.9–7002.6)	NS
S2 (%)	14.3 (14.0–17.3)	15.0 (10.7–21.2)	NS
S2 (pmol/mg)	2328.8 (1605.7–2622.0)	2565.0 (1358.1–5405.3)	NS

**Table 5:** Comparison of the percentage and concentration in each IgG4 N-glycan between patients with IgG4-related disease with or without kidney disease.

	Kidney disease (+) n = 6 Median (range)	Kidney disease (−) n = 6 Median (range)	p value
G0 (%)	39.3 (30.7–48.2)	36.0 (35.0–44.6)	NS
G0 (pmol/mg)	7079.9 (5702.5–9210.5)	5617.9 (4039.6–7817.3)	NS
G1 (%)	22.6 (21.6–24.1)	24.5 (22.9–27.2)	NS
G1 (pmol/mg)	3963.7 (3240.0–6065.8)	4006.6 (2724.5–5036.2)	NS
G2 (%)	36.5 (28.6–45.5)	38.2 (27.2–40.6)	NS
G2 (pmol/mg)	5963.8 (4132.5–12763.2)	6072.6 (3505.8–8925.2)	NS
G3 (%)	0.5 (0.0–1.0)	0.3 (0.0–1.1)	NS
G3 (pmol/mg)	82.9 (0.0–198.4)	32.0 (0.0–251.2)	NS
F0 (%)	3.6 (2.6–6.5)	4.8 (2.0–9.6)	NS
F0 (pmol/mg)	710.4 (448.8–942.5)	734.0 (394.8–1537.1)	NS
F1 (%)	96.4 (93.5–97.4)	95.2 (90.4–98.0)	NS
F1 (pmol/mg)	16,557.6 (13,592.5–27,255.3)	14602.9 (10,324.5–21,205.5)	NS
S0 (%)	61.8 (52.8–67.2)	61.1 (54.9–69.7)	NS
S0 (pmol/mg)	10,737.9 (9620.3–15,631.6)	9148.3 (7009.4–12,251.5)	NS
S1 (%)	23.2 (19.5–26.1)	23.4 (19.5–27.5)	NS
S1 (pmol/mg)	3793.7 (2835.0–7002.6)	3996.6 (2432.1–5552.0)	NS
S2 (%)	15.0 (13.3–21.2)	14.3 (10.7–19.9)	NS
S2 (pmol/mg)	2452.4 (1930.0–5405.3)	2433.6 (1358.1–4374.0)	NS

**Table 6:** Comparison of the percentage and concentration in each IgG4 N-glycan between patients with IgG4-related disease with or without lymphadenopathy.

	Lymphadenopathy (+) n = 7 Median (range)	Lymphadenopathy (−) n = 5 Median (range)	p value
G0 (%)	36.1 (32.8–48.2)	36.4 (30.7–44.7)	NS
G0 (pmol/mg)	7204.8 (5322.8–9210.5)	5641.9 (4039.6–7698.2)	NS
G1 (%)	23.8 (21.6–24.3)	24.7 (22.5–27.2)	NS
G1 (pmol/mg)	4048.8 (3240.0–6065.8)	3878.6 (2724.5–4667.2.2)	NS
G2 (%)	39.3 (28.6–45.5)	37.5 (27.7–45.5)	NS
G2 (pmol/mg)	6201.3 (4132.5–12763.2)	5542.9 (3505.8–9337.7)	NS
G3 (%)	0 (0.0–1.1)	0.5 (0.0–1.0)	NS
G3 (pmol/mg)	0 (0.0–251.2)	81.7 (0.0–198.4)	NS
F0 (%)	2.8 (2.0–6.5)	5.9 (3.5–9.6)	NS
F0 (pmol/mg)	696.2 (394.8–942.5)	724.6 (626.8–1537.1)	NS
F1 (%)	97.2 (93.5–98.0)	94.1 (90.4–96.5)	NS
F1 (pmol/mg)	16,531.2 (13,592.5–27,255.3)	14,445.1 (10,324.5–19,782.0)	NS
S0 (%)	61.0 (54.9–67.2)	63.5 (52.8–69.7)	NS
S0 (pmol/mg)	10,651.2 (8952.8–15,631.6)	9343.9 (7009.4–11,160.7)	NS
S1 (%)	23.7 (19.5–25.2)	22.0 (19.5–27.5)	NS
S1 (pmol/mg)	3854.4 (2835.0–7002.6)	3637.5 (2432.1–5342.6)	NS
S2 (%)	15.4 (13.3–19.9)	14.0 (10.7–21.2)	NS
S2 (pmol/mg)	2622.0 (1930.0–5405.3)	2245.1 (1358.1–4339.3)	NS

## Discussion

This study reports significant increases of G0 IgG4 glycan concentrations in IgG4RD compared with healthy controls. Furthermore, F1 IgG4 glycan levels in IgG4RD were significantly increased in IgG4RD compared with healthy control. However, there were no significant differences in the levels of sialic acid or IgG4 glycan with bisecting arm between IgG4RD and healthy controls. Furthermore, we observed a significant decrease in F0 IgG4 glycans in IgG4RD with hypocomplementemia compared with those without hypocomplementemia, although the difference was small. There were no significant differences in the galactosylation and sialyation of IgG4 glycans between IgG4RD with and without hypocomplementemia. Regarding the individual organ involvement of IgG4RD (kidney, pancreas, lymph node), there were no significant differences in the galactosylation, fucosylation, and sialyation of IgG4 glycans between patients with and without each organ involvement.

Recent studies strongly suggest that G0 glycans have proinflammatory nature[36, 37, 42]. For example, IgGs with G0 glycans increased their affinity for FcγRIII, an activating FcγR. Although we observed an increase of G0 IgG4 glycans in patients with IgG4RD, and the proinflammatory nature of G0 glycans has been reported in a number of inflammatory diseases, it is not clear whether agalactosylated IgG4 is involved in the pathogenesis of IgG4RD because IgG4 has a low binding ability to FcγR. Furthermore, recent studies suggest that IgG4 has an anti-inflammatory rather than proinflammatory role.

IgG subclasses other than IgG4 have proinflammatory properties that induce activation of the complement system as well as stimulating phagocytic or cytotoxic reactions. Unlike other IgG subclasses, IgG4 does not have the same proinflammatory properties, because IgG4 does not bind to C1q compared with FcγRI, RII, and RIIII[43]. Furthermore, IgG4 can exchange half its molecules resulting in the formation of chimeric antibodies with two different binding specificities, which are monovalent and unable to cross-link identical antigens to form ICs[44–46]. Therefore, IgG4 is presumed to have an anti-inflammatory rather than a proinflammatory role.

However, although IgG4 has anti-inflammatory properties, it does not mean that IgG4 is not pathogenic. Several reports showed that IgG4 autoantibodies were implicated in the pathogenesis of autoimmune diseases without requiring complement or other immune components. Huijhers et al reported that IgG4 autoantibodies to muscle-specific kinase cause myasthenia gravis by inhibiting the binding between muscle specific kinase and lipoprotein receptor-related protein[47, 48]. In pemphigus, IgG4 anti-desmoglein antibodies directly disrupt the epithelial layer and cause blister formation without complement activation or antibody-dependent cell-mediated cytotoxicity. Furthermore, IgG4 antibodies that recognize disintegrin-like and metalloproteinase with thrombospondin type 1 motifs 13 (ADAMTS13) are involved in the pathogenesis of thrombotic thrombocytopenic purpura[49–51]. Regarding IgG4RD, autoantibodies to putative antigens have been reported in patients with IgG4-related autoimmune pancreatitis and sialoadenitis. However, these autoantibodies have also been reported in other autoimmune diseases indicating a lack of disease-specificity for IgG4RD[52]. Currently, the pathogenic role of these autoantibodies has not been proven.

Elevated levels of G0 IgG were reported in the serum of patients with granulomatosis with polyangiitis (GPA, formerly named Wegener’s granulomatosis)[29]. Interestingly, 31% of biopsy specimens showed increased IgG4-positive cells in patients with GPA, thus GPA may mimic IgG4RD histologically[53]. Furthermore, in patients with GPA, IgG4 antibodies were reported to play a role in its pathogenesis. Holland et al. reported that IgG4 proteinase 3-antineutrophil cytoplasmic antibodies (PR3-ANCA) isolated from the sera of GPA patients activated neutrophils resulting in the production of superoxide. As the activity of stimulating neutrophils was independent of the neutrophil expression of FcγRI, this suggested that PR3-ANCA IgG4 antibodies activated neutrophils through FcγRIIa and FcγRIIIb[54]. Furthermore, Hussain et al. showed that monoclonal IgG4 anti PR3 antibodies induced the dose-dependent release of superoxide from neutrophils, as well as neutrophil degranulation and adhesion[55]. These reports suggest that IgG4 anti-PR3 may participate in the pathogenesis of vasculitis in GPA by stimulating neutrophils to induce a proinflammatory response. Taken together, G0-IgG4 in patients with IgG4RD may participate in the pathogenesis of IgG4RD by the same mechanism as GPA, whereby neutrophils are stimulated followed by the induction of proinflammatory responses.

In this study, we observed a significant increase in F1 glycan concentration. Recently, Gornik et al. reported the increased level of serum IgG with F1 glycan in patients with RA[56]. Furthermore, Sjöwall et al. showed that patients with SLE had a significant increased exposure to fucosyl residues of complexed IgG in their serum[57]. Although it is unclear whether fucosylated IgG participates in the pathogenesis of these diseases, Sjöwall et al. hypothesized that highly fucosylated site of complexed IgG molecules may facilitate their uptake by binding to lectin receptors on the phagocyte surface to induce chronic inflammation. Further study on the pathogenic role of F1 IgG4 glycans in patients with IgG4RD is required.

In this study, although there were no significant differences, the absolute concentration of G1, G2, and F0 glycans tended to be higher in IgG4RD than in healthy controls; thus, the total concentrations of all IgG4 glycans were increased in IgG4RD. As a result, the percentages of G0 as well as F1 glycans were lower and there was no significant difference between IgG4RD and healthy controls. Thus, the increase in total IgG4 glycans might be caused by increased amounts of Fab glycans. Because we used whole IgG4 as a sample, we detected N-glycans including the Fc and Fab portion of IgG4. Recent studies revealed that between 15%–25% of the total IgG glycome originates from Fab[58, 59], and that Fab-linked glycans have less core fucose, and more galactose and sialic acid compared with Fc-linked glycans[60–62]. Therefore, we think that an increase in the extent of Fab glycosylation might explain the lack of a significant difference in the proportion of IgG4 G0 glycans as well as F1 glycans in the total glycome of IgG4RD. Furthermore, compared with Fc glycan, Fab glycan is highly sialylated, and S1 glycan is the most abundant compared with S0 and S2 glycans[62]. Therefore, an increase in the extent of Fab glycosylation might affect the significantly elevated level of S1IgG4 glycan in IgG4RD compared with healthy controls, despite no significant changes in S0 and S2IgG4 glycans.

Recent studies show that agalactosylated IgG acts as an epitope for complement-activating mannose-binding lectins resulting in activation of the lectin complement pathway[37]. Furthermore, in addition to activation of the lectin complement pathway, IgGs with G0 glycans also activate the classical and alternative complement pathways[63]. Therefore, we expected that hypogalactosylated IgG4 may contribute to hypocomplementemia in patients with IgG4RD. However, we did not observe significant differences in the galactosylation and sialyation of IgG4 glycans between IgG4RD with and without hypocomplementemia. Instead, we observed significant differences in the fucosylation of IgG4 glycans between IgG4RD with and without hypocomplementemia, although the difference was small. In general, afucosylated IgG abrogates binding C1q and therefore the antibody’s capacity to activate the complement system. Interestingly, a recent study showed that exposure to fucosyl residues on circulating IgG immune complexes was significantly correlated with the consumption of serum C3 in patients with SLE. However, IgG4 does not generally bind to C1q and does not activate the complement pathway. One possibility is that IgG4 isolated from IgG4RD with hypocomplementemia binds to C1q and is associated with the consumption of serum C3. Another possibility is that decreased levels of IgG4 F0 glycans and the increased tendency of IgG4 F1 glycans did not reflect complement activation directly, but rather chronic inflammation caused by complement activation indirectly. Further study is needed to determine the contribution of the IgG4 glycome to the mechanism of complement activation in IgG4RD with hypocomplementemia.

Regarding the individual organ involvement of IgG4RD (kidney, pancreas, lymph node), there were no significant differences in the galactosylation, fucosylation, and sialyation of IgG4 glycans between patients with and without each organ involvement, which may suggest that the altered glycome of IgG4 is not related to the differences observed for individual organ involvement.

Although the elevation of agalactosylated IgG levels are associated with several inflammatory and autoimmune diseases, the cause of the increase is not well understood. One possibility might be that reduced B-cell galactosyltransferase activity leads to lower galactosylation. Axford et al reported an association between reduced galactosyltransferase activity and serum agalactosylated IgG level in RA[64, 65]. Another possibility is that microenvironmental factors present during the differentiation of B cells to antibody-secreting cells might influence the glycosylation of IgG. Recent studies reported that the microenvironmental factors CpG oligodeoxynucleotide and interleukin-21 increased Fc-linked galactosylation and reduced bisecting N-acetylglucosamine levels, whereas all-trans retinoic acid (a natural metabolite of vitamin A) significantly decreased galactosylation and sialylation levels[66–68].

This study had several limitations. First, the numbers of patients and controls were relatively small because of our research budget. Second, it was impossible to differentiate N-glycans released from the Fab and Fc portions of IgG4. We used whole IgG4 as samples; therefore, we detected N-glycans from the Fc portion and the Fab portion of IgG4. Finally, because the number of IgG4RD patients and healthy controls were different, their age and gender were not matched. The proportion of agalactosylated IgG was reported to increase with age[69]. Although the proportion (%) of agalactosylated IgG between IgG4RD and healthy controls was not significantly different in this study, we cannot exclude the possibility that increased levels of G0 IgG4 glycans may be related to aging.

Although we observed changes in IgG4 glycosylation isolated from the sera of patients with IgG4RD compared with healthy controls, it is not clear whether the increased IgG4 is the cause or a consequence of the inflammatory reactions associated with IgG4RD. Further studies of IgG4RD are needed to clarify the mechanism of IgG4 production and the role of IgG4 in the pathogenesis of IgG4RD.

## Supporting information

S1 Fig SDS-PAGE analysis of isolated IgG4. Original uncropped gel.
(TIF)

S2 Fig Western blot analysis of anti-IgG1 antibody reacting with isolated IgG4. Original uncropped blot.
(TIF)

S3 Fig Western blot analysis of anti-IgG2 antibody reacting with isolated IgG4. Original uncropped blot.
(TIF)

S4 Fig Western blot analysis of anti-IgG3 antibody reacting with isolated IgG4. Original uncropped blot.
(TIF)

S5 Fig Western blot analysis of anti-IgG4 antibody reacting with isolated IgG4. Original uncropped blot.
(TIF)

S1 Data Participant-level data. (XLSX)
