# Supplementary figures and images for: Changes in N-glycans of IgG4 and its relationship with the existence of hypocomplementemia and individual organ involvement in patients with IgG4-related disease
Source: PLoS One. 2018 Apr 19;13(4):e0196163. doi: 10.1371/journal.pone.0196163 (PMC5908088; doi:10.1371/journal.pone.0196163)

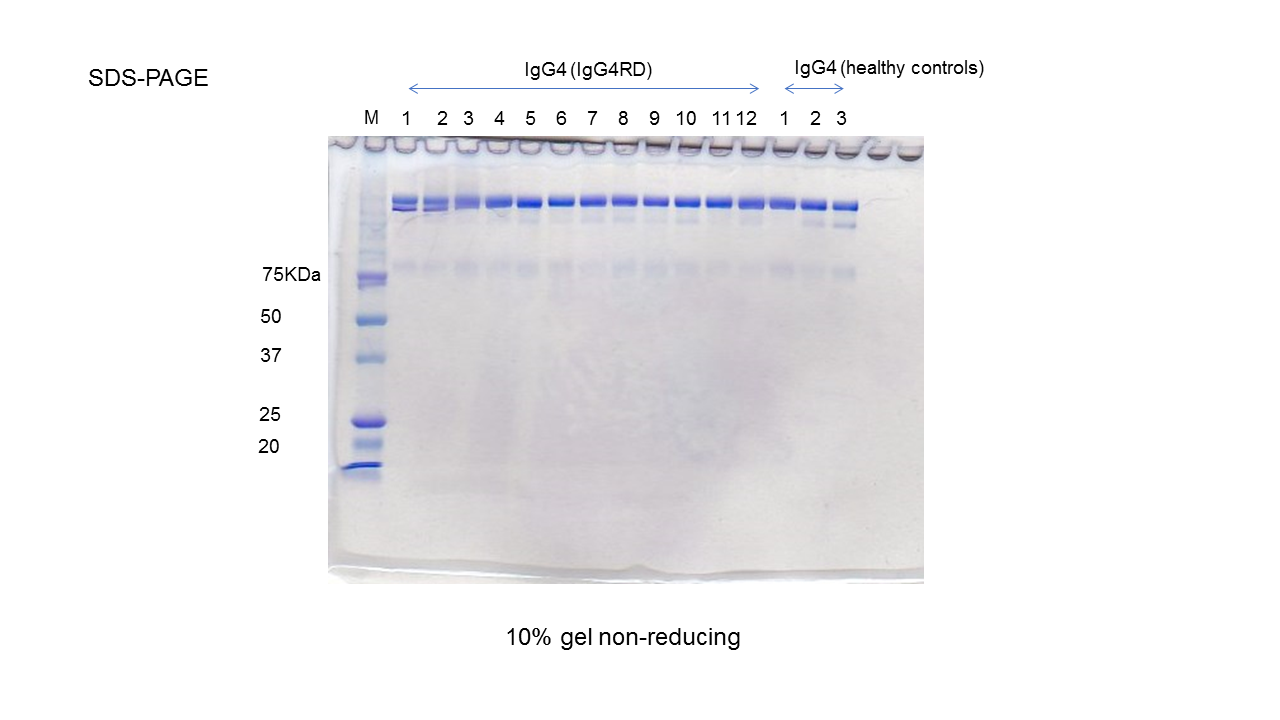

Supplement: S1 Fig — Original uncropped gel. (TIF) [file pone.0196163.s001.TIF]

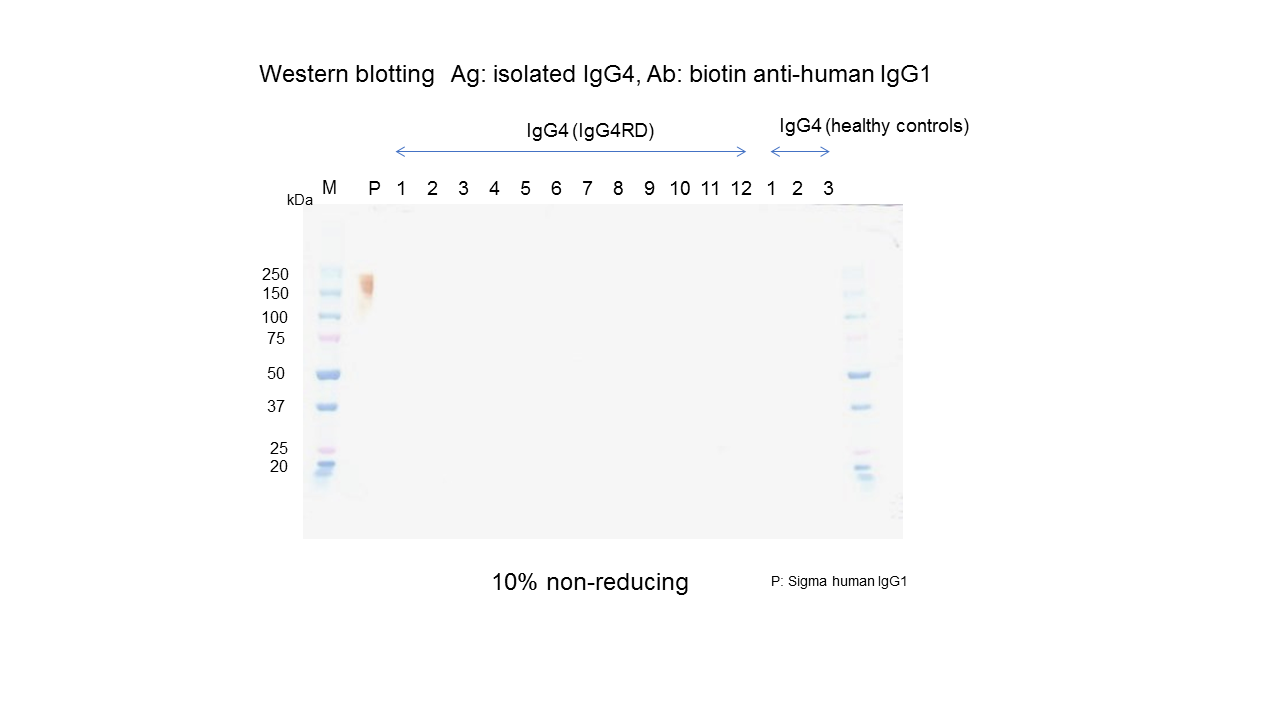

Supplement: S2 Fig — Original uncropped blot. (TIF) [file pone.0196163.s002.TIF]

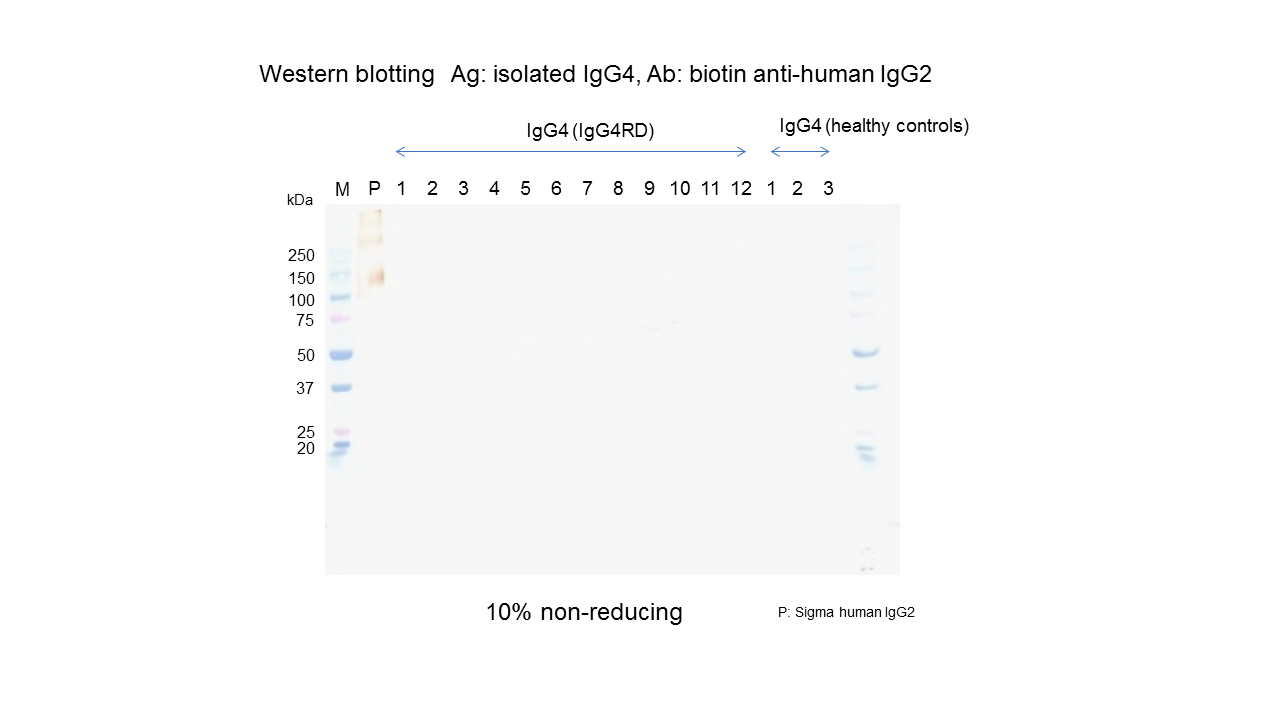

Supplement: S3 Fig — Original uncropped blot. (TIF) [file pone.0196163.s003.TIF]

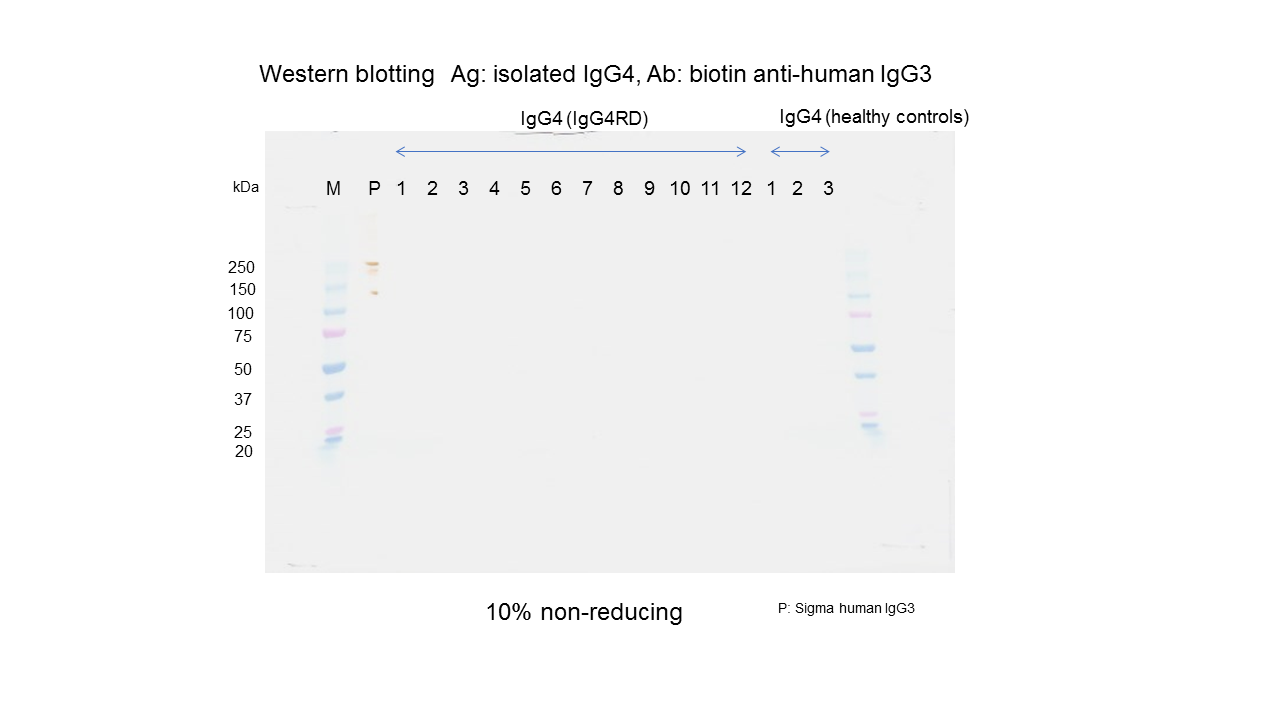

Supplement: S4 Fig — Original uncropped blot. (TIF) [file pone.0196163.s004.TIF]

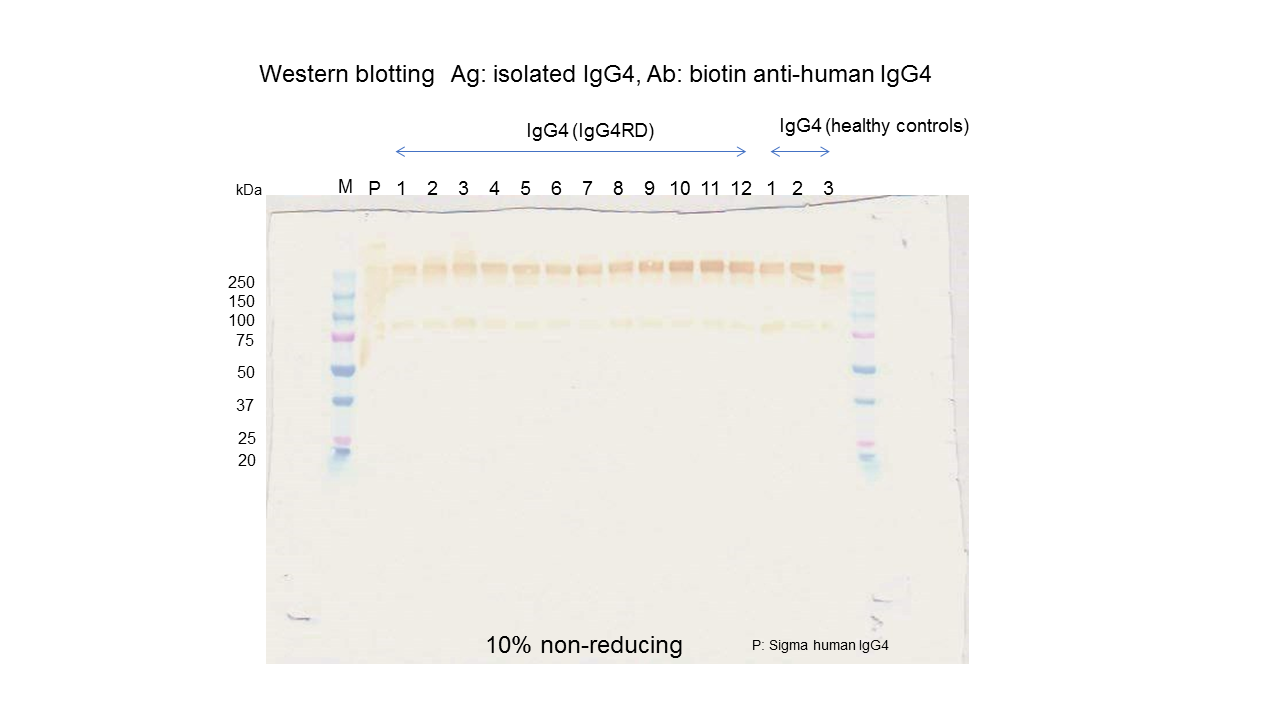

Supplement: S5 Fig — Original uncropped blot. (TIF) [file pone.0196163.s005.TIF]
